# Phase II study of vincristine, actinomycin-D, cyclophosphamide and irinotecan for patients with newly diagnosed low-risk subset B rhabdomyosarcoma: A study protocol

**DOI:** 10.1097/MD.0000000000018344

**Published:** 2019-12-27

**Authors:** Mitsuru Miyachi, Kunihiko Tsuchiya, Ako Hosono, Atsushi Ogawa, Katsuyoshi Koh, Atsushi Kikuta, Junichi Hara, Satoshi Teramukai, Hajime Hosoi

**Affiliations:** a Department of Pediatrics, Graduate School of Medical Science, Kyoto Prefectural University of Medicine, Kyoto; b Division of Pediatric Oncology, National Cancer Center Hospital East, Kashiwa, Chiba; c Department of Pediatrics, Niigata Cancer Center Hospital, Niigata; d Department of Hematology/Oncology, Saitama Children's Medical Center, Saitama; e Department of Pediatric Oncology, Fukushima Medical University Hospital, Fukushima; f Department of Pediatric Hematology/Oncology, Children's Medical Center, Osaka City General Hospital, Osaka; g Department of Biostatistics, Graduate School of Medical Science, Kyoto Prefectural University of Medicine, Kyoto, Japan.

**Keywords:** cyclophosphamide, irinotecan, low-risk, microRNA, rhabdomyosarcoma, UGT1A1

## Abstract

**Background:**

Approximately 80% to 90% of patients with low-risk rhabdomyosarcoma can be cured. However, cured patients often face long-term complications associated with the treatment. An important factor in the treatment plan is the dose of cyclophosphamide administered because the dose can have both acute and long-term side effects. It is therefore essential to investigate whether the dose can be reduced without a negative effect on treatment outcome. The ARST0331 trial revealed that drastically reducing the cyclophosphamide dose to 4.8 g/m^2^ negatively affected treatment outcomes. The current study aims to determine whether reducing the cyclophosphamide dose to 10.8 g/m^2^ while introducing a new drug, irinotecan, can prevent the negative effect on treatment outcome. We also aim to investigate whether the reduced cyclophosphamide dose results in a decrease in infertility, one of the long-term complications of this treatment.

**Methods:**

The subjects are patients with stage 1 group III rhabdomyosarcoma (excluding those with orbital group III N0 and NX) or patients with stage 3 group I and II low-risk subset B embryonal rhabdomyosarcoma who will alternately undergo VAC 1.2 treatment (vincristine, actinomycin D, cyclophosphamide 1.2 g/m^2^) and VI treatment (vincristine, irinotecan). The effectiveness and safety of this treatment regimen will be assessed. Data will be presented at international conferences and will be published in peer-reviewed journals.

**Discussion:**

This study is significant because it aims to establish that the use of irinotecan in patients with low-risk subset B embryonal rhabdomyosarcoma (aged 30 or younger) allows the dose of cyclophosphamide to be reduced and is associated with few short-term adverse effects and long-term complications. The open-label and single-arm design of this study may be a limitation.

**Trial registration and ethical approval:**

The trial registration number is jRCTs051180200 (Japan Registry of Clinical Trials). The study protocol was approved by the institutional review board at each of the participating centers and the data will be presented at international conferences and published in peer-reviewed journals.

## 1 Introduction

Rhabdomyosarcoma is a malignant tumor that originates in the mesoderm or mesenchymal tissue of the fetus and develops during the ensuing formation of skeletal muscles or during skeletal muscle differentiation following malignant transformation. Treatment consists of a combination of chemotherapy and radiotherapy after tumor resection or biopsy.

In the low-risk subset B patients followed in the JRS-I trial (conducted in Japan between 2004 and 2012), an experimental protocol was utilized that consisted of 24 weeks of VAC (vincristine, actinomycin D, cyclophosphamide) therapy and 24 weeks of VA (vincristine, actinomycin D) therapy. Enrollment closed in March 2012 and analysis of the data is on-going, but no clear declines in treatment outcomes were observed. However, the dose of cyclophosphamide used in each cycle of the 24-week VAC therapy + 24-week VA therapy for JRS-I low-risk subset B patients was as high as 2.2 g/m^2^. The total amount of the drug that was administered during the treatment cycle was 17.6 g/m^2^, which is high enough to cause known acute toxicities such as veno-occlusive disease and late effects such as infertility.

Since most rhabdomyosarcoma patients in the low-risk group can expect long-term survival due to curative treatment of the disease, late effects are not desired. To this end, studies on lower doses of cyclophosphamide have been conducted in the United States. The ARST0331 trial, conducted by the Soft Tissue Sarcoma (STS) committee of the Children's Oncology Group (COG), utilized four cycles of VAC 1.2 regimen, each cycle of which consists of 1.2 g/m^2^ of cyclophosphamide (total cyclophosphamide dose of 4.8 g/m^2^) and 4 cycles of VA regimen. However, their results showed that the estimated 3-year failure-free survival (FFS) rate was 63% (95% CI: 46%, 75%), and the overall survival (OS) rate was 84% (95% CI: 68%, 93%),^[1]^ which were unsatisfactory outcomes.

Based on the above, the present study will alternately utilize nine cycles of VAC 1.2 treatment (cyclophosphamide dose of 10.8 g/m^2^) and five cycles of VI treatment, thus modestly reducing the dose of cyclophosphamide. According to statistics released by the Surveillance, Epidemiology, and End Results (SEER) program,^[2]^ 40% of the patients diagnosed with rhabdomyosarcoma are adults. This study is therefore significant because it aims to establish a treatment for low-risk subset B rhabdomyosarcoma patients (aged 30 years or younger) that has few short-term adverse effects and long-term complications without leading to poorer therapeutic outcomes.

Irinotecan was therapeutically effective in 70% of patients when used in combination with vincristine in the COG STS Phase II Window Trial D9802 of high-risk rhabdomyosarcoma patients; moreover, of 50 patients, the drug lad to a complete response in one patient and a partial response in 34 patients.^[3]^ This response rate was higher than that reported in other Phase II trials of rhabdomyosarcoma, and the rate demonstrated that the treatment had the same efficacy as VAC when administered to untreated metastatic rhabdomyosarcoma patients.

Interestingly, adults experience higher frequencies of diarrhea and neutropenia owing to irinotecan because of *UGT1A1* gene polymorphisms.[^4^,^5^] In Japanese adults, the *UGT1A1*∗28 and *UGT1A1*∗6 gene polymorphisms are associated with irinotecan-induced diarrhea and neutropenia.[^6^,^7^] However, pediatric studies conducted in the United States did not determine an association between the *UGT1A1*∗28 gene polymorphism and adverse effects of irinotecan, such as diarrhea and neutropenia.[^8^,^9^]

It remains unknown whether there is an association between *UGT1A1* gene polymorphisms (*UGT1A1*∗28, *UGT1A1*∗6) and irinotecan-induced diarrhea and neutropenia in Japanese children. The current study also aims to elucidate the association between the frequency of adverse events associated with the combined use of vincristine and irinotecan and *UGT1A1* gene polymorphisms.

Although rhabdomyosarcoma is the commonest form of STS in children, there is currently no tumor marker that can be measured in standard blood tests, making preoperative diagnosis difficult. In contrast, the presence of tumor remnants following initial surgery is a predictive factor for poor prognosis. If a preliminary diagnosis of rhabdomyosarcoma can be made prior to initial surgery, it would be possible to make a preoperative plan with the objective of total resection. Thus, the presence of a minimally invasive biomarker is important to the achievement of preoperative diagnosis of rhabdomyosarcoma. In recent years, studies have confirmed the presence of non-coding RNA, such as microRNA, that is not translated to protein. The expression profiles of some microRNAs have been reported to be specific to certain tissues and tumors.[^10^,^11^] A study on cell lines reported that in cases of pediatric cancer, the expression profiles differed from tumor to tumor.^[12]^ Another study further explored this difference and revealed that blood serum test results for rhabdomyosarcoma showed elevated expression of muscle-specific *miR-206*.^[13]^ However, that study utilized a small sample size. Validation of their results in studies with larger numbers of subjects is therefore required.

This study aims to verify the validity of the use of serum *miR-206* as a biomarker to estimate therapeutic response and prognosis.

## 2 Methods

### 2.1 Study aims

The objective of this clinical trial is to assess the efficacy and safety of alternating use of VAC 1.2 treatment (vincristine, actinomycin D, cyclophosphamide) and VI treatment (vincristine, irinotecan) on low-risk subset B embryonal rhabdomyosarcoma patients classified as Stage 1, Group III (excluding orbital Group III N0 and NX) or Stage 3, Group I and II.

### 2.2 Study design

This study is an open-label, single-arm, multicenter phase II trial that will be performed at 100 centers in Japan. The primary endpoint is event-free survival (EFS). Secondary endpoints include overall survival, time to treatment failure, overall response rate, frequency of adverse events, and grade assessment using CTCAE ver. 4.0. Exploratory endpoints include the correlation between serum *miR-206* expression level at diagnosis and EFS, changes in *miR-206* expression level during and after treatment, and the correlation between the expression level of *miR-206* expression in cerebrospinal fluid at the time of diagnosis of parameningeal primary tumor and central nervous system recurrence. Other exploratory endpoints are the frequency of adverse effects of irinotecan and the quality control of radiotherapy.

### 2.3 Patient registration and data collection

Patient registration started on February 1, 2016 and the target enrolment is 18 patients. A log with the patients’ names and dates of birth will be kept along with their unique study numbers at each participating center. All the data generated from the study will be stored in an anonymized form in a database, which will also be password protected. Only anonymized information will be stored on this, and participants will only be identifiable by their study number. All paperwork will be kept in a locked filing cabinet in a locked office. All data will be stored on a password-protected computer with limited access to the research team.

### 2.4 Eligibility criteria

Inclusion criteria

1) Diagnosis of low-risk subset B rhabdomyosarcoma. Patients switched from other risk groups according to the results of central pathology review are acceptable.
2) Age: Under 30 years of age at the time consent was obtained.
3) Start of treatment within 42 days after the date of initial surgery.
4) Initial onset of malignant tumor.
5) Patients determined to have the following performance status (PS) according to the Eastern Cooperative Oncology Group (ECOG) Performance Status Score:
  PS 0–2
  In cases of were aged 15 or younger at the time consent was obtained, the standard is a Lansky performance status score of at least 50 points.
6) Patients whose major organ functions have been preserved. Maintenance of the following: WBC of at least 2000/μl, neutrophil count of at least 500/μl, hemoglobin of at least 7.0 g/dL, platelet count of at least 50,000/μl.
7) Patients for whom a consent form was obtained from the patient him/herself or from a legally authorized representative (in the case of patients aged between 16 and 20 years who have been informed of their diagnosis, the consent must be obtained from the patient and his/her parents).

Exclusion criteria

1) Primary tumor in the central nervous system, central nervous system metastasis, or positive cerebrospinal fluid cytology.
2) Active multiple cancer (synchronous multiple cancer and metachronous multiple cancer with a disease-free period of 5 or fewer years).
3) Demyelinating Charcot-Marie-Tooth disease or varicella zoster.
4) History of chemotherapy and radiotherapy.
5) The following severe concurrent diseases:
  o Interstitial pneumonia, pulmonary fibrosis, or advanced emphysema;
  o Poorly controlled diabetes;
  o Poorly controlled hypertension;
  o Disease with marked ECG abnormality or clinical problems (cardiac failure, myocardial infarction, ischemic disease);
  o Cirrhosis of the liver, liver failure;
  o Renal failure.
6) Presence of contraindications for the treatment drugs.
7) Any of the following:
  o Women who are or may be pregnant or women hoping to become pregnant;
  o Women who are breastfeeding;
  o Men whose partners hope to become pregnant during the treatment period;
8) Any other reason determined by the principal investigator or secondary investigators not eligible for the study.

### 2.5 Treatment methods (Figure 1)

**Figure 1:**
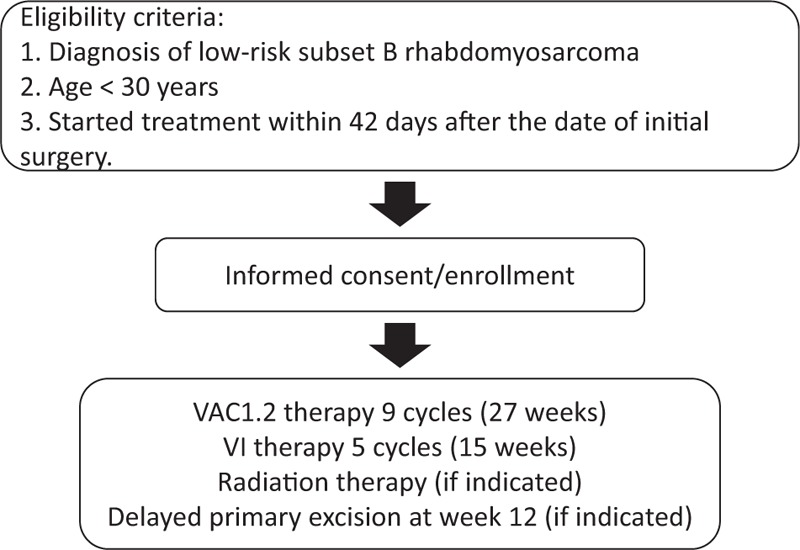
Study flow chart.

Subgroup B patients will receive 9 cycles (27 weeks) of vincristine, dactinomycin, and cyclophosphamide at 1.2 g/m^2^/cycle (VAC1.2) and 5 cycles (15 weeks) of vincristine and irinotecan (VI) (total cumulative doses: V = 54 mg/m^2^, A = 0.405 mg/kg, C = 10.8 g/m^2^, I = 1250 mg/m^2^) (Fig. 1).

Radiotherapy (RT) will begin at week 13 for most patients with residual microscopic or gross embryonal rhabdomyosarcoma. Patients in group II/III will receive RT as follows: 36 Gy for stage 1 group IIA patients, 41.4 Gy for group IIB/IIC patients, and 50.4 Gy for group III patients.

Two exceptions will be made. Subgroup B patients with tumors in the vulva, uterus, biliary tract, and superficial non-parameningeal head/neck sites can undergo a delayed primary excision (DPE) at week 12 to remove gross residual tumor. RT will be administered after the DPE. Subgroup B patients with vaginal tumors will begin RT at week 12 if N1 or at week 28 if N0, in an attempt to preserve vaginal tissue and to avoid RT; N0 patients in whom repeated biopsies showed no residual tumor before or by week 28 will receive no RT.

### 2.6 Follow-up

The planned blood tests and imaging studies will be performed at enrollment, at 12 weeks, at 24 weeks, and at the conclusion of the treatment. Imaging studies will additionally be performed every three months following the conclusion of treatment.

### 2.7 Study endpoints and statistical methods

The primary endpoint is event-free survival (EFS). EFS is defined as the period from the enrollment date until disease worsening/recurrence or death from any cause. The EFS curve will be estimated using the Kaplan-Meier estimator, and the null hypothesis will be tested using a test based on the maximum likelihood estimator of hazard. When one sided *P* value for this test is below .05 (equivalent to a 3-year EFS rate higher than 78%), it will be determined that the EFS in the treatment group was superior to that reported by ARST0331. Sub-group analysis will be performed by estimating the EFS curve by age, postoperative group, tissue type, presence of fusion gene, and number of copies of serum *miR-206* at diagnosis (high/low).

The target sample size is 18 patients. Assuming that the enrollment rate will remain constant during the enrollment period, all cases will be completely followed, and the distribution of EFS will be the exponential distribution. The 3-year EFS rate of the ARST0331 (63%) is used as a threshold. When the expected 3-year EFS rate for this treatment is 85% (event hazard = 0.054/year), the number of subjects required in order to reject the null hypothesis of 3-year EFS rate (63%; event hazard = 0.154/year) at a one-sided significant level of 5% and a power of 80%, using a test based on the maximum likelihood estimator for hazard is 18 patients.

Analysis of secondary endpoints:

1) Overall survival (OS)
  The OS curve will be estimated using the Kaplan–Meier estimator.
2) Tumor response
  The response rate will be estimated by the proportion for complete response (CR) or partial response (PR).
3) Adverse events
  Data on adverse events will be tabulated by type.

Analysis of exploratory endpoints:

1) We will assess the correlation between *miR-206* expression level at diagnosis and EFS, changes in serum *miR-206* expression during and after treatment, and *miR-206* expression levels in cerebrospinal fluid in cases of parameningeal primary tumor and central nervous system recurrence. The Kaplan–Meier estimator will be used to estimate the EFS curve by serum number of *miR-206* copies (high/low) at diagnosis. The frequencies of subsequent central nervous system recurrence by number of *miR-206* copies (high/low) at diagnosis will be compared.
2) In order to assess the correlation between the frequency of *UGT1A1* gene polymorphisms and the adverse effects of irinotecan, data will be compiled on the incidence of diarrhea and neutropenia associated with the presence vs absence of *UGT1A1* gene polymorphisms (∗6, ∗28).

## 3 Discussion

Rhabdomyosarcoma is the most common type of soft-tissue tumor in childhood. This study will determine whether a cyclophosphamide dose of 10.8 g/m^2^, administered alongside irinotecan, is an effective and less toxic treatment for low-risk subset B rhabdomyosarcoma patients. The data from this study will also elucidate whether circulating *miR-206* can serve as a novel prognostic biomarker for rhabdomyosarcoma.

## Author contributions

All authors were members of the protocol planning working group and contributed to the design of the study. MM is a principal investigator of the study. MM drafted the manuscript. All authors critically reviewed and approved the final version of the manuscript.
